# Associations between lean mass and leptin in men with chronic spinal cord injury: Results from the FRASCI-muscle study

**DOI:** 10.1371/journal.pone.0198969

**Published:** 2018-06-27

**Authors:** Andrew J. Park, Ricardo A. Battaglino, Nguyen M. H. Nguyen, Leslie R. Morse

**Affiliations:** 1 Department of Physical Medicine and Rehabilitation, University of Colorado School of Medicine, Aurora, Colorado, United States of America; 2 Rocky Mountain Regional Spinal Injury System, Craig Rehabilitation Hospital, Englewood, Colorado, United States of America; Augusta University, UNITED STATES

## Abstract

Leptin is an adipo-myokine that regulates appetite and energy expenditure by a neuroendocrine feedback loop. Leptin levels are positively correlated with BMI in the spinal cord injury population and leptin levels are greater in individuals with spinal cord injury compared to uninjured controls. Leptin is produced in multiple tissues, including fat, bone, and skeletal muscle and is a putative biomarker of sedentary behavior in older adults. We assessed body composition leptin, adiponectin, and IL-6 levels in 205 men with chronic spinal cord injury. We found no association between age, injury duration, injury level, injury completeness, or walking status and leptin. There was a significant positive association between lean mass and leptin in men with SCI that was independent of fat. Adjusting for body composition, leptin levels were positively associated with IL-6 and negatively associated with adiponectin levels. When considering men with SCI and sarcopenic obesity, only fat mass remained positively associated with leptin. We found no association between IL-6, adiponectin, or lean mass and leptin in the sarcopenic obesity group. Our findings suggest that lean mass is an under recognized, but substantial, source of circulating leptin. Furthermore, SCI-related sarcopenic obesity may result in dysregulated adipo-myokine metabolism with local and systemic physiologic effects.

## Introduction

Leptin has classically been identified as an adipokine produced by adipocytes that regulates weight balance and energy expenditure by a neuroendocrine feedback loop between adipose tissue and the hypothalamus [1]. Transgenic mice lacking leptin receptor isoforms consistently demonstrate an obese phenotype with significantly more adipose tissue and less lean mass compared to wild type mice [2]. In human studies, leptin levels are positively correlated with obesity in the general population [3] and positively associated with sedentary behavior, even after adjusting for various possible confounding factors including demographics, medications, and body mass index (BMI) [4–6]. However, the frail elderly with high prevalence of low lean mass defined as sarcopenia have low leptin levels and higher leptin levels are associated with increased longevity in centenarians, suggesting a role for leptin in skeletal muscle metabolism [7].

Leptin receptors are abundant in human skeletal muscle [8]. Leptin is produced by and regulates skeletal muscle directly through myoblast leptin receptors in an autocrine fashion [2, 9–14] and through a central neuroendocrine pathway that is mediated by insulin-like growth factor 1 (IGF-1) [15–16]. An elegant *in vivo* study demonstrated that skeletal muscle produces leptin and that the per unit mass of leptin release from adipose tissue is only slightly greater than skeletal muscle in humans[9]. The endocrine function of muscle has been studied extensively and has led some to use the term “adipo-myokine” for cytokines that are produced in both muscle and fat and signal in an autocrine or paracrine manner, such as leptin and interleukin-6 (IL-6). As skeletal muscle represents a greater total body composition percentage than adipose tissue, skeletal muscle may play a greater role in leptin production and regulation than previously appreciated. These findings suggest that muscle may be an important source of circulating leptin and that muscle disorders, including atrophy and sarcopenia, may impact the autocrine functions of muscle-derived leptin.

Obesity, sedentary behavior, and sarcopenia are all prevalent after spinal cord injury (SCI). Several studies have reported that people with SCI have higher leptin levels than non-injured controls [10, 17–22]. These results are consistent with known body composition changes that occur after SCI, including increased total fat mass and lower lean mass [23]. Obesity increases the production and release of pro-inflammatory adipokines, including leptin and IL-6. This occurs with a simultaneous reduction of anti-inflammatory adipokines, including adiponectin. The impact of this shift in balance between pro- and anti-inflammatory cytokines on muscle-fat interactions is poorly understood and there is limited information on these interactions following SCI. Therefore, in this study we sought to assess the association between circulating adipo-myokines and lean mass in men with chronic SCI.

## Materials and methods

### Subjects

**Fig 1:**
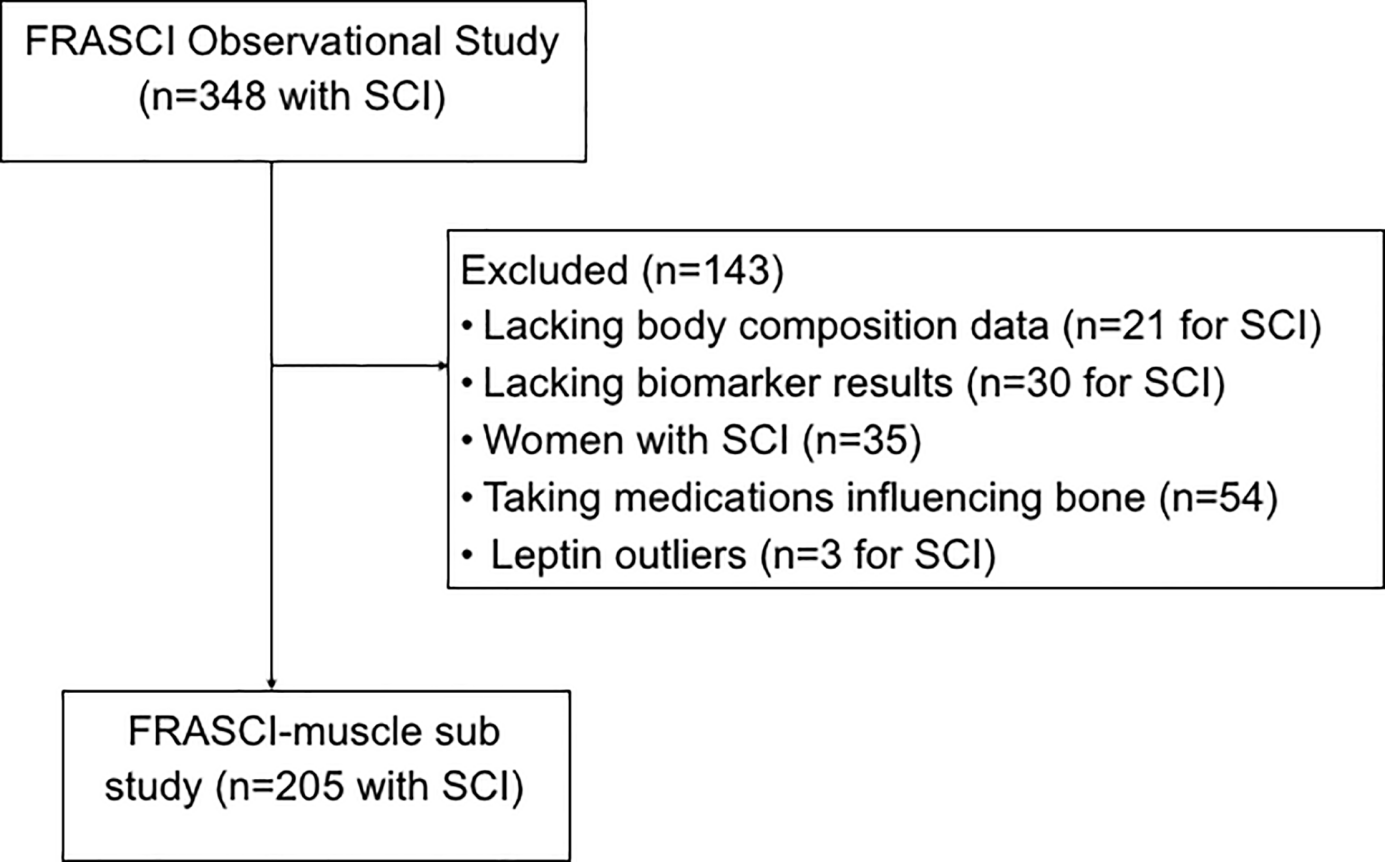
FRASCI-muscle cohort. The final cohort for this muscle sub-study (FRASCI-muscle).

For this muscle sub study, we assessed participants with chronic SCI who were enrolled in the longitudinal Fracture Risk after SCI (FRASCI) Study. Study inclusion criteria and recruitment methods for the parent cohort study have previously been described [24–25]. Briefly, participants with SCI were eligible if they were 22 years of age or older, one or more years after injury, were not ventilator dependent, did not have a tracheostomy, and had no other neuromuscular disease. 348 participants with SCI were enrolled in this cohort between August 2009 and December 2014 and completed testing. We excluded 51 subjects because body composition (n = 21) or biomarker results (n = 30) were not available. We excluded women with SCI (n = 35), as there were too few to make meaningful comparisons based on gender. We also excluded 54 participants actively taking medications known to influence bone metabolism [bisphosphonates (n = 23), warfarin (n = 16), hormones (n = 10), bisphosphonate + warfarin (n = 3), bisphosphonate + hormone (n = 2)]. 3 participants (72,175.3–122,358.5 pg/mL) excluded based on leptin levels that were considered to be outliers. The final cohort for this muscle sub study (FRASCI-muscle) consisted of 205 men with SCI (Fig 1). The Institutional Review Boards approved all protocols prior to initiation of the study, and all participants gave their written informed consent to participate.

### Motor score

Motor level and completeness of injury were confirmed by physical exam at study entry by a trained rater according to the American Spinal Injury Association Impairment Scale (AIS). Participants were classified as AIS A or B (motor complete, no motor function below the neurological level of injury); AIS C (motor incomplete, motor function preserved below the neurological level, and more than half the key muscles below the neurological level are not strong enough to overcome gravity); or AIS D (motor incomplete, motor function preserved below the neurological level, and more than half the key muscles below the neurological level strong enough to overcome gravity). Injury severity was then classified in 2 categories: motor complete SCI (AIS A/B) or motor incomplete SCI (AIS C or D).

### Dual X-ray absorptiometry (DXA) for body composition

We used a 5th generation GE Healthcare iDXA dual x-ray absorptiometry (DXA) scanner with enCore configuration version 12.3 to assess body composition. Total fat mass (kg) and total lean mass (kg) were calculated by the system software from whole body scans based on body weight measured at the time of scanning. As a standard procedure, a quality assurance phantom supplied by the manufacturer was measured at least every 2 days to confirm accuracy of the densitometer.

### Biochemical analyses

Subjects were asked to undergo testing in a fasting state and efforts were made to collect samples in the morning before a meal. For subject safety, individuals were advised to have a light meal or snack if fasting could worsen a medical condition (orthostatic hypotension). In all cases information was collected on time since last meal or snack. Plasma samples were drawn into an EDTA tube and immediately delivered to the core blood research laboratory at our facility. The samples were centrifuged for 15 min at 2600 rpm (1459 x g) at 4°C and stored at -80°C until batch analysis. All biochemical analyses were performed at the Clinical & Epidemiologic Research Laboratory, Department of Laboratory Medicine at Children’s Hospital in Boston, a state-of-the-art reference laboratory that specializes in micro-analysis. Leptin was measured by ultra-sensitive enzyme linked immunosorbent assay (ELISA) (R & D Systems, Minneapolis, MN) with a sensitivity of 7.8 pg/mL and day-to-day variability of 5.4, 4.2 and 3.5% at concentrations of 65.7, 146 and 581 pg/mL, respectively. Total adiponectin was measured by ELISA (ALPCO Diagnostics Inc., Salem, NH) with a detection limit of 0.075 ng/ml. and day-to-day variability less than 15% at various concentrations for all forms of adiponectin. Interleukin-6 (IL-6) was determined by ultra-sensitive ELISA (R & D Systems, Minneapolis, MN) with a sensitivity of 0.094 pg/ml and day-to-day variability of 9.6, 7.2 and 6.5% at concentrations of 0.49, 2.78 and 5.65 pg/mL, respectively. Assays were performed in duplicate and any duplicate with >10% CV was repeated.

### Variable definition

Information regarding SCI, medical history, and medication use was obtained by questionnaire at the time of DXA scan. Participants were weighed and supine length measured for the calculation of body mass index (BMI). In subjects with severe joint contractures, length was self-reported (n = 14). Usual mobility mode (more than 50% of the time) was considered in the following 2 categories: wheelchair use (motorized wheelchair or hand-propelled wheelchair) or walking (with aid such as crutch, cane or walk without assistance). Obesity was defined as having a BMI ≥ 25 for SCI [26,27]. Sarcopenia was defined as having an appendicular lean mass index ≤ 7.26 [27]. Sarcopenic-obesity was defined as being sarcopenic (ALMI ≤ 7.26) and having total body % fat ≥ 25 [26,27]. For body composition total lean mass (kg) was included in the analyses.

### Statistical analysis

All analyses were performed using SAS 9.4 (SAS Institute, Inc., Cary, NC). T-tests or χ^2^ tests were used to compare subject characteristics as appropriate. General linear models (PROC GLM) were applied to assess associations between leptin and lean mass. Factors with a *p* value of <0.10 in the univariate models, as well as factors that were deemed clinically significant (age), were included in the multivariable models assessing the association of lean mass and leptin (PROC GLM). Factors with a *p* value of <0.05 were considered statistically significant and any factor with a *p* value of >0.05 was removed from the models.

## Results

### Subject characteristics

Subject characteristics are presented in Table 1. All participants were male and the majority white. Ages ranged from 22.7 to 85.7 years with a mean of 54.3 ± 13.7. Injury duration ranged from 4.7 to 30.7 years with a mean of 17.7 ± 13.0 years. Nearly 60% of participants used a wheelchair as their primary mobility mode with the majority (72%) using manual wheelchairs. A majority of the subjects were obese (68%), had sarcopenia (31%), and/or had sarcopenic-obesity (27%). A majority of subjects (79%) had not consumed anything for at least 8 hours prior to testing. Leptin, adiponectin, and IL-6 levels did not vary significantly based on time since last meal or snack (p = 0.41 for leptin, p = 0.14 for adiponectin, and p = 0.45 for IL-6).

**Table 1:** FRASCI-muscle cohort participant characteristics.

Variable	(n = 205)
**Age** (years) [Mean ± SD]	54.3 ± 13.7
**White** (n%)	172 (83.9)
**Years post injury** [Mean ± SD]	17.7 ± 13.0
**BMI** (kg/m^2^) [Mean ± SD]	27.7 ± 5.4
**Total fat mass** (%) [Mean ± SD]	35.7 ± 7.8
**Total lean Mass** (kg) [Mean ± SD]	53.5 ± 8.9
**ASIA level**	
Motor complete:	
A/B, n(%)	92 (44.9)
Motor incomplete:	
C, n(%)	17 (8.3)
D, (n%)	96 (46.8)
**Wheelchair users,** n(%)	120 (58.5)
Motorized, n(%)	34 (28.3)
Manual, n(%)	86 (71.7)
**Tetraplegia**, n(%)	100 (48.8)
**Obese**, n(%)	140 (68.3)
**Sarcopenic**, n(%)	63 (30.7)
**Sarcopenic-obesity**, n(%)	56 (27.3)
**Leptin** (pg/mL) [Mean ± SD]	13,229.7 ± 11,051.2
**Adiponectin** (ng/ml) [Mean ± SD]	4,916.4 ± 2,724.2
**IL-6** (ng/ml) [Mean ± SD]	3.5 ± 4.0

### Clinical factors associated with ln leptin levels

In univariate analyses leptin levels were positively associated with injury duration, BMI, fat mass, total lean mass, IL-6, and obesity status and were negatively associated with adiponectin and sarcopenia status (Table 2). Age, walking status, tetraplegia vs paraplegia did not reach significance. In multivariate models that included all men with SCI (Table 3), ln leptin was negatively associated with ln adiponectin (p = 0.001) and positively associated with total lean mass, total fat mass, and ln IL-6 (p = 0.001-<0.0001). Leptin levels increased by 1.09 pg/mL for every 1% increase in fat mass and by 1.02 pg/mL for every kilogram increase in lean mass. This model explained 73% of the variation in ln leptin. These relationships remained unchanged in multivariable models restricted to men with SCI and no sarcopenic-obesity (p = 0.001-<0.0001). However, when limiting the analysis to men with SCI and sarcopenic obesity, only total fat mass remained positively associated with ln leptin (p = <0.0001). Leptin levels also increased by 1.08 pg/mL for every 1% increase in fat mass in this group. We found no significant association between ln leptin and lean mass or ln adiponectin (p = 0.27–0.30). There was a positive association between ln IL-6 and ln leptin that trended toward significance (p = 0.06). This model explained 73% of the variation in ln leptin.

**Table 2:** Univariate factors associated with ln leptin in men with chronic SCI.

	SCI (n = 205)	
Variable	β ± SE	p
**Age** (years)	0.009 ± 0.005	0.07
**Injury duration** (years)	0.01 ± 0.005	0.03
**BMI** (kg/m^2^)	0.12 ± 0.008	<0.0001
**Total fat mass** (%)	0.095 ± 0.004	<0.0001
**Total lean mass** (kg)	0.03 ± 0.007	<0.0001
**ln adiponectin (**ng/ml)	-0.63 ± 0.11	<0.0001
**ln IL-6 (**ng/ml)	0.51 ± 0.06	<0.0001
**Walking status**		
Wheelchair user	0.08 ± 0.13	0.57
Walk with or without aid	reference	
**Injury completeness**		
Motor complete	0.06 ± 0.13	0.65
Motor incomplete	reference	
**Injury level**		
Tetraplegia	-0.13 ± 0.13	0.31
Paraplegia	reference	
**Obesity status**		
Obese	1.25 ± 0.11	<0.0001
Not obese	reference	
**Sarcopenia status**		
Sarcopenia	-0.37 ± 0.14	0.008
No sarcopenia	reference	
**Sarcopenic-obesity status**		
Sarcopenic-obesity	-0.12 ± 0.15	0.41
No sarcopenic-obesity	reference	

**Table 3:** Multivariable model of factors associated with ln leptin in men with SCI and based on sarcopenic-obesity status.

	All SCI (n = 205)			No Sarcopenic obesity (n = 149)			Sarcopenic obesity (n = 56)		
	p<0.0001, R^2^ = 0.73			p<0.0001, R^2^ = 0.75			p<0.0001, R^2^ = 0.73		
Variable	β ± SE	e^β^	p	β ± SE	e^β^	p	β ± SE	e^β^	p
**Total fat mass** (%)	0.08 ± 0.004	1.08	<0.0001	0.09 ± 0.006	1.09	<0.0001	0.09 ± 0.009	1.09	<0.0001
**Total lean mass (kg)**	0.02 ± 0.003	1.02	<0.0001	0.02 ± 0.005	1.02	0.001	0.01 ± 0.01	1.01	0.30
**ln adiponectin**	-0.21 ± 0.06	0.81	0.001	-0.23 ± 0.08	0.79	0.005	-0.15 ± 0.13	0.86	0.27
**ln IL-6**	0.14 ± 0.04	1.15	0.001	0.16 ± 0.05	1.17	0.003	0.15 ± 0.08	1.16	0.06

## Discussion

We examined body composition and circulating levels of leptin in 205 men with chronic SCI. We found no association between age, injury duration, injury level, injury completeness, or walking status and leptin. There was a significant positive association between lean mass and leptin in men with SCI that was independent of fat. Adjusting for body composition, leptin levels were positively associated with IL-6 and negatively associated with adiponectin levels. When considering men with SCI and sarcopenic obesity, only fat mass remained positively associated with leptin. We found no association between IL-6, adiponectin, or lean mass and leptin in the sarcopenic obesity group.

Our findings suggest that lean mass contributes independently to circulating leptin levels in men with SCI with normal body composition. These results suggest sarcopenia leads to impaired leptin production and/or release from skeletal muscle following SCI. In the current study nearly one third of the men with SCI were sarcopenic and 27% had sarcopenic-obesity. These results are consistent with a previous study demonstrating high prevalence of sarcopenic obesity in adults with SCI [26]. Extreme muscle wasting begins immediately with a 33% reduction in thigh cross-sectional area within 3 months after SCI, and occurs with both increased intramuscular fat accumulation and increased central adiposity [28]. It is possible that intramuscular fat is a significant source of muscle-derived leptin. Our findings of lower leptin levels in those with sarcopenia versus those with normal muscle mass suggests that intramuscular fat is not the primary source of muscle-derived leptin. However, our study design did not include assessments of intramuscular fat. Future studies focused on associations between intramuscular fat and circulating leptin are needed to test this hypothesis.

Crosstalk between muscle and adipose tissue is poorly understood, but it is well documented in both mouse and human studies that leptin receptors are abundant in high concentrations in skeletal muscle and that skeletal muscle produces leptin [8,9,11,29]. Leptin, recently identified as an adipo-myokine, may link the metabolic rate of skeletal muscle to fat mass and therefore nutrient availability. Indeed, coordinated muscle-adipose metabolism has been reported in several studies [30–33]. Moreover, a high-fat diet increases leptin expression in both skeletal muscle and adipose tissue [29,34]. Leptin, therefore, likely plays a critical role in maintaining balance between adipose tissue and skeletal muscle mass.

Previous literature commonly credits increased central adiposity as the cause of elevated leptin levels after SCI without considering the contributions of skeletal muscle [35]. Nonetheless, perpetual elevation of circulating leptin levels may have multiple consequences on the central neuroendocrine pathway. Leptin is well known to participate in energy homeostasis by interaction with the long form leptin receptor LepRb which actives Jak2/Stat3 pathway in the arcuate nucleus of the hypothalamus. This ultimately triggers various signaling cascades in metabolism including suppression of feeding behavior [36–39]. After prolonged activation of the LepRb receptor, the downstream activation of the SOCS3 pathway appears to attenuate LepRb receptors leading to leptin resistance [36]. Chronic SCI leads to reduction in LepRb and Jak2/Stat3 signaling and additionally increases expression of SOCS3, consistent with a central leptin resistance after SCI [37]. Additionally, persistent down stream signaling results in attenuation of anorexigenic neuroendocrine pathways and disinhibition leading to pro-orexigenic effects. Persistent activation of these pathways likely contribute to metabolic dysfunction [38]. Paradoxically, elevated leptin levels appear to have the opposite effect on peripheral leptin receptor expression following SCI. Leptin receptors and downstream pro-inflammatory pathways are significantly increased in several visceral organs, including pancreatic and cardiac tissue [38]. This is particularly relevant given the high prevalence of cardiovascular disease after SCI [35]. This divergent leptin expression and regulation from central and peripheral tissues has previously been described as selective leptin resistance, although a mechanism for these differences has not yet been identified [39].

The impact of SCI-induced sarcopenia on leptin signaling within skeletal muscle and systemically is unknown. It is also unclear if muscle-derived leptin and fat-derived leptin have unique targets in their local environments, in the periphery, or centrally. We do know that obesity and exercise both influence the balance of pro-inflammatory and anti-inflammatory cytokines including leptin [40–42], and that distinct leptin receptor isoforms have been described in human skeletal muscle and adipose tissue [8]. Additionally, differences in co-expression of cytokines in skeletal muscle versus adipose tissue may result in distinct local signaling milieus. The ratio of leptin to adiponectin is positively associated with muscle strength in older adults [22] and is a biomarker of atherosclerotic disease, insulin resistance, and metabolic syndrome in the general population [43]. Similarly, IL-6 is produced by both contracting skeletal muscle and adipose tissue and regulates adipogenesis and production of adiponectin [44]. It has been suggested that obesity-related inflammatory cytokines, including tumor necrosis factor α (TNFα), interleukin-1β (IL-1β) and IL-6, may accelerate muscle catabolism [45–47]. Muscle loss does not appear to plateau in chronic SCI [48], suggesting that fat-mediated mechanisms compound the effects of mechanical unloading on muscle atrophy. In the current study, leptin levels were negatively associated with adiponectin levels and positively associated with IL-6, and this is consistent with prior reports [10]. Interestingly, there was no longer a significant association between leptin and IL-6 or adiponectin in men with SCI and sarcopenic obesity. These findings support the concept of dysregulated adipo-myokine activity in men with SCI with sarcopenic obesity.

## Conclusion

Skeletal muscle mass is positively associated with leptin in men with chronic SCI and normal body composition. The development of SCI-induced sarcopenic obesity disrupts muscle/leptin associations suggesting dysregulated adipo-myokine activity. The systemic consequences of abnormal skeletal muscle-derived leptin production and/or release are unclear and warrant further investigation.
